# Tetra­kis(μ-naphthalene-1-acetato-κ^2^ *O*:*O*′)bis­[(*N*,*N*-dimethyl­formamide-κ*O*)copper(II)]

**DOI:** 10.1107/S1600536812007064

**Published:** 2012-02-29

**Authors:** Fu-Jun Yin, Yu Gu, Hong Zhao, Di-Si Bai

**Affiliations:** a Jiangsu Marine Resources Development, Research Institute, Huaihai Institute of Technology, Lianyungang 222005, People’s Republic of China; b Qian’an College, Hebei United University, Tangshan 063009, People’s Republic of China; c Department of Chemical Engineering, Huaihai Institute of Technology, Lianyungang 222005, People’s Republic of China

## Abstract

The asymmetric unit of the title compound, [Cu_2_(C_12_H_9_O_2_)_4_(C_3_H_7_NO)_2_], contains two independent centrosymmetric dinuclear copper(II) complexes. The central paddle-wheel units are formed by four bridging bidentate naphthalene-1-acetate ligands with two dimethyl­formamide ligands in the axial positions. The unique Cu^II^ ions have slightly distorted square-pyramidal coordination geometries. One of the naphthalene rings is disordered over two sets of sites, with refined occpancies of 0.535 (4) and 0.465 (4).

## Related literature

For coordination compounds of 1-naphthyl­acetate, see: Yin *et al.* (2010 ▶); Chen *et al.* (2004 ▶); Yang *et al.* (2008 ▶); Xia *et al.* (2006 ▶); Ji *et al.* (2011 ▶).
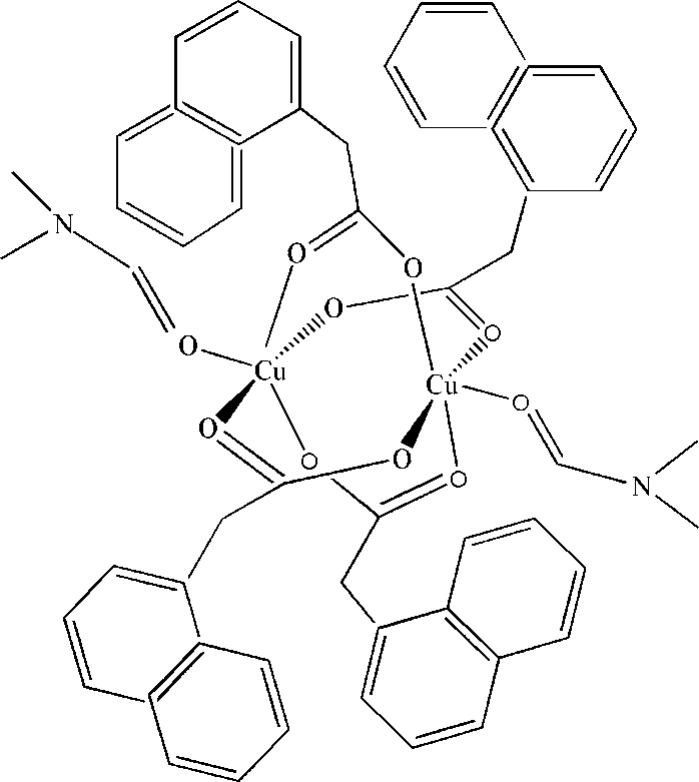


## Experimental

### 

#### Crystal data

[Cu_2_(C_12_H_9_O_2_)_4_(C_3_H_7_NO)_2_]
*M* *_r_* = 1014.04
Triclinic, 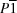

*a* = 10.6704 (7) Å
*b* = 12.3561 (8) Å
*c* = 20.7734 (14) Å
α = 74.8390 (11)°
β = 84.898 (1)°
γ = 66.848 (1)°
*V* = 2430.3 (3) Å^3^
*Z* = 2
Mo *K*α radiation
μ = 0.94 mm^−1^
*T* = 298 K
0.10 × 0.10 × 0.10 mm

#### Data collection

Bruker APEXII CCD diffractometer
Absorption correction: multi-scan (*SADABS*; Sheldrick, 1996 ▶) *T*_min_ = 0.945, *T*_max_ = 0.945
18538 measured reflections
8516 independent reflections
7103 reflections with *I* > 2σ(*I*)
*R*_int_ = 0.019

#### Refinement

*R*[*F* ^2^ > 2σ(*F* ^2^)] = 0.043
*wR*(*F* ^2^) = 0.115
*S* = 1.04
8516 reflections
630 parameters
16 restraints
H-atom parameters constrained
Δρ_max_ = 0.61 e Å^−3^
Δρ_min_ = −1.25 e Å^−3^

### 

Data collection: *APEX2* (Bruker, 2007 ▶); cell refinement: *SAINT* (Bruker, 2007 ▶); data reduction: *SAINT*; program(s) used to solve structure: *SHELXS97* (Sheldrick, 2008 ▶); program(s) used to refine structure: *SHELXL97* (Sheldrick, 2008 ▶); molecular graphics: *SHELXTL* (Sheldrick, 2008 ▶); software used to prepare material for publication: *SHELXTL*.

## Supplementary Material

Crystal structure: contains datablock(s) I, global. DOI: 10.1107/S1600536812007064/lh5416sup1.cif

Structure factors: contains datablock(s) I. DOI: 10.1107/S1600536812007064/lh5416Isup2.hkl

Additional supplementary materials:  crystallographic information; 3D view; checkCIF report

## Figures and Tables

**Fig. 1.**
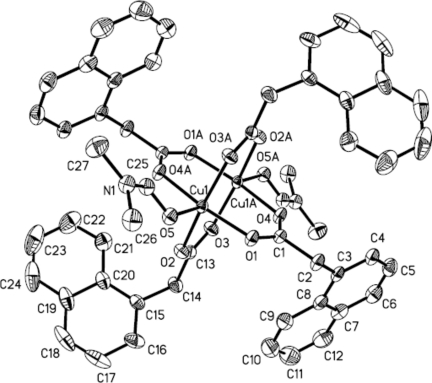
The molecular structure of one of the independent molecules of (I) showing displacement ellipsoids at the 30% probability [symmetry code (A): -x+2, -y+1, -z+1].

**Fig. 2.**
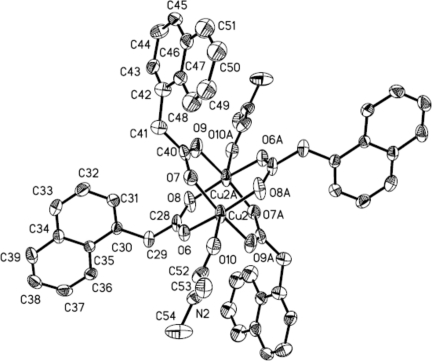
The molecular structure of the other independent molecule of (I) showing displacement ellipsoids at the 30% probability. [symmetry code (A): -x+1, -y+2, -z].

## supplementary crystallographic information

### Comment

Naphthalene-1-yl-acetate acid is a plant-growth regulator and exhibits remarkable coordination versatility towards metal cations (Yin *et al.*, 2010; Chen *et al.*, 2004; Yang *et al.*, 2008; Xia *et al.*,2006; Ji *et al.*, 2011). In continuation of the structural studies of metal complexes of this ligands, the crystal structure of the title compound was determined.

The molecular structure of the title compound (I) (see Figs. 1 & 2), contains a centrosymmetric dinuclear copper paddle-wheel unit, in which each Cu^II^ ion is coordinated by four O atoms from a naphthalen-1-yl-acetate ligand in the basal plane and one O atom of a *N*,*N*-dimethylformamide ligand in the axial position to form a square-pyramidal coordination geometry. Both independent molecules lie on crystallographic inversion centers.

### Experimental

The title compound was synthesized by the reaction of Cu(NO_3_)_2_ × 3 H_2_O (72.3 mg, 0.3 mmol), naphthalene-1-yl-acetic acid (93 mg, 0.5 mmol), and NaOH (20 mg, 0.5 mmol) in 10 ml of *N*,*N*-dimethylformamide under solvothermal conditions. The mixture was homogenized and transferred into a sealed Teflon-lined solvothermal bomb (volume: 25 ml) and heated to 423K for three days. After cooling green crystals of the title compound were obtained, which were washed with distilled water and absolute ethanol (yield: 48.5% based on Cu(NO_3_)_2_ × 3 H_2_O).

### Refinement

H atoms were placed in calculated positions, with C—H = 0.93 or 0.96 Å and included in the final cycles of refinement using a riding model with *U*_iso_(H) = 1.2 or 1.5*U*_eq_(C). One of the naphthalene rings is disordered over two sets of sites with refined occpancies of 0.535 (4) and (0.465 (4).

### Crystal data

**Table:**

[Cu_2_(C_12_H_9_O_2_)_4_(C_3_H_7_NO)_2_]	*Z* = 2
*M**_r_* = 1014.04	*F*(000) = 1052
Triclinic, *P*1	*D*_x_ = 1.386 Mg m^−^^3^
Hall symbol: -P 1	Mo *K*α radiation, λ = 0.71073 Å
*a* = 10.6704 (7) Å	Cell parameters from 7990 reflections
*b* = 12.3561 (8) Å	θ = 2.2–26.9°
*c* = 20.7734 (14) Å	µ = 0.94 mm^−^^1^
α = 74.8390 (11)°	*T* = 298 K
β = 84.898 (1)°	Block, green
γ = 66.848 (1)°	0.10 × 0.10 × 0.10 mm
*V* = 2430.3 (3) Å^3^	

### Data collection

**Table:**

Bruker APEXII CCD diffractometer	8516 independent reflections
Radiation source: fine-focus sealed tube	7103 reflections with *I* > 2σ(*I*)
Graphite monochromator	*R*_int_ = 0.019
φ and ω scans	θ_max_ = 25.0°, θ_min_ = 2.1°
Absorption correction: multi-scan (*SADABS*; Sheldrick, 1996)	*h* = −12→12
*T*_min_ = 0.945, *T*_max_ = 0.945	*k* = −14→14
18538 measured reflections	*l* = −24→24

### Refinement

**Table:**

Refinement on *F*^2^	Primary atom site location: structure-invariant direct methods
Least-squares matrix: full	Secondary atom site location: difference Fourier map
*R*[*F*^2^ > 2σ(*F*^2^)] = 0.043	Hydrogen site location: inferred from neighbouring sites
*wR*(*F*^2^) = 0.115	H-atom parameters constrained
*S* = 1.04	*w* = 1/[σ^2^(*F*_o_^2^) + (0.0486*P*)^2^ + 2.352*P*] where *P* = (*F*_o_^2^ + 2*F*_c_^2^)/3
8516 reflections	(Δ/σ)_max_ = 0.001
630 parameters	Δρ_max_ = 0.61 e Å^−^^3^
16 restraints	Δρ_min_ = −1.25 e Å^−^^3^

### Special details

**Table:**

Geometry. All e.s.d.'s (except the e.s.d. in the dihedral angle between two l.s. planes) are estimated using the full covariance matrix. The cell e.s.d.'s are taken into account individually in the estimation of e.s.d.'s in distances, angles and torsion angles; correlations between e.s.d.'s in cell parameters are only used when they are defined by crystal symmetry. An approximate (isotropic) treatment of cell e.s.d.'s is used for estimating e.s.d.'s involving l.s. planes.
Refinement. Refinement of *F*^2^ against ALL reflections. The weighted *R*-factor *wR* and goodness of fit *S* are based on *F*^2^, conventional *R*-factors *R* are based on *F*, with *F* set to zero for negative *F*^2^. The threshold expression of *F*^2^ > σ(*F*^2^) is used only for calculating *R*-factors(gt) *etc*. and is not relevant to the choice of reflections for refinement. *R*-factors based on *F*^2^ are statistically about twice as large as those based on *F*, and *R*- factors based on ALL data will be even larger.

### Fractional atomic coordinates and isotropic or equivalent isotropic displacement parameters (Å^2^)

**Table:**

	*x*	*y*	*z*	*U*_iso_*/*U*_eq_	Occ. (<1)
Cu1	0.86554 (3)	0.53893 (3)	0.496213 (16)	0.03371 (10)	
O1	0.8617 (2)	0.69486 (17)	0.50264 (11)	0.0474 (5)	
O2	0.8686 (2)	0.48613 (19)	0.59443 (10)	0.0488 (5)	
O3	1.0954 (2)	0.4191 (2)	0.60003 (10)	0.0505 (5)	
O4	1.0886 (2)	0.62875 (17)	0.50813 (11)	0.0478 (5)	
O5	0.65309 (19)	0.59391 (19)	0.47337 (11)	0.0481 (5)	
N1	0.5038 (2)	0.5742 (2)	0.41095 (13)	0.0501 (6)	
C1	0.9708 (3)	0.7076 (2)	0.50813 (14)	0.0396 (6)	
C2	0.9582 (3)	0.8313 (3)	0.51355 (18)	0.0518 (8)	
H2A	0.9535	0.8829	0.4690	0.062*	
H2B	0.8734	0.8676	0.5355	0.062*	
C3	1.0737 (3)	0.8278 (3)	0.55139 (17)	0.0491 (7)	
C4	1.1703 (3)	0.8671 (3)	0.51830 (19)	0.0573 (8)	
H4	1.1640	0.8955	0.4721	0.069*	
C5	1.2789 (4)	0.8656 (3)	0.5526 (2)	0.0696 (10)	
H5	1.3437	0.8925	0.5290	0.083*	
C6	1.2896 (4)	0.8254 (4)	0.6193 (2)	0.0724 (11)	
H6	1.3610	0.8263	0.6414	0.087*	
C7	1.1946 (4)	0.7819 (3)	0.6562 (2)	0.0652 (9)	
C8	1.0849 (3)	0.7827 (3)	0.62141 (19)	0.0558 (8)	
C9	0.9902 (4)	0.7392 (4)	0.6595 (2)	0.0755 (11)	
H9	0.9184	0.7383	0.6375	0.091*	
C10	1.0003 (6)	0.6988 (5)	0.7266 (3)	0.1008 (15)	
H10	0.9356	0.6716	0.7502	0.121*	
C11	1.1078 (7)	0.6980 (5)	0.7602 (3)	0.1063 (17)	
H11	1.1148	0.6701	0.8064	0.128*	
C12	1.2028 (5)	0.7377 (5)	0.7261 (3)	0.0910 (14)	
H12	1.2746	0.7356	0.7494	0.109*	
C13	0.9805 (3)	0.4372 (3)	0.62552 (14)	0.0429 (7)	
C14	0.9800 (4)	0.3984 (4)	0.70118 (16)	0.0592 (9)	
H14E	1.0662	0.3317	0.7160	0.071*	
H14F	0.9765	0.4655	0.7182	0.071*	
C15	0.8679 (3)	0.3594 (3)	0.73282 (15)	0.0514 (8)	
C16	0.7770 (5)	0.4238 (4)	0.77268 (18)	0.0784 (12)	
H16A	0.7809	0.4954	0.7772	0.094*	
C17	0.6777 (5)	0.3840 (6)	0.8070 (2)	0.111 (2)	
H17A	0.6164	0.4294	0.8339	0.133*	
C18	0.6706 (5)	0.2804 (6)	0.8014 (3)	0.106 (2)	
H18A	0.6056	0.2541	0.8253	0.127*	
C19	0.7591 (4)	0.2122 (4)	0.7603 (2)	0.0781 (13)	
C20	0.8604 (3)	0.2511 (3)	0.72557 (16)	0.0559 (8)	
C21	0.9501 (4)	0.1789 (3)	0.6852 (2)	0.0743 (11)	
H21A	1.0179	0.2020	0.6620	0.089*	
C22	0.9383 (7)	0.0752 (4)	0.6798 (3)	0.115 (2)	
H22A	0.9979	0.0288	0.6529	0.138*	
C23	0.8379 (10)	0.0380 (6)	0.7143 (4)	0.146 (3)	
H23A	0.8312	−0.0328	0.7103	0.176*	
C24	0.7526 (7)	0.1037 (6)	0.7525 (4)	0.119 (2)	
H24A	0.6861	0.0780	0.7750	0.143*	
C25	0.6245 (3)	0.5422 (3)	0.43674 (16)	0.0478 (7)	
H25A	0.6915	0.4723	0.4233	0.057*	
C26	0.3898 (3)	0.6778 (3)	0.4237 (2)	0.0679 (10)	
H26A	0.4112	0.7001	0.4610	0.102*	
H26B	0.3109	0.6574	0.4336	0.102*	
H26C	0.3712	0.7447	0.3850	0.102*	
C27	0.4771 (4)	0.5066 (4)	0.3696 (2)	0.0817 (12)	
H27A	0.5601	0.4409	0.3641	0.122*	
H27B	0.4427	0.5597	0.3267	0.122*	
H27C	0.4108	0.4747	0.3908	0.122*	
Cu2	0.38926 (5)	0.97711 (5)	0.01857 (2)	0.07018 (17)	
O6	0.3889 (3)	1.0570 (3)	0.08925 (12)	0.0702 (7)	
O7	0.5200 (3)	0.8234 (3)	0.07183 (12)	0.0721 (7)	
O8	0.5772 (3)	1.0939 (3)	0.05852 (13)	0.0949 (11)	
O9	0.7071 (3)	0.8613 (3)	0.04101 (13)	0.0897 (10)	
O10	0.2111 (3)	0.9402 (3)	0.05243 (13)	0.0763 (8)	
N2	0.0108 (3)	0.9822 (3)	0.10633 (17)	0.0696 (8)	
C28	0.4779 (4)	1.0960 (4)	0.09576 (17)	0.0685 (10)	
C29	0.4625 (5)	1.1507 (4)	0.15480 (17)	0.0715 (10)	
H2B1	0.3675	1.2023	0.1582	0.086*	
H2B2	0.5151	1.2014	0.1471	0.086*	
C30	0.5091 (3)	1.0557 (3)	0.22023 (16)	0.0516 (8)	
C31	0.5883 (4)	0.9376 (4)	0.22208 (19)	0.0686 (10)	
H4B	0.6157	0.9151	0.1821	0.082*	
C32	0.6299 (4)	0.8485 (4)	0.2826 (2)	0.0802 (12)	
H5B	0.6840	0.7684	0.2823	0.096*	
C33	0.5912 (4)	0.8797 (4)	0.3410 (2)	0.0753 (11)	
H6B	0.6193	0.8205	0.3808	0.090*	
C34	0.5097 (4)	0.9990 (3)	0.34265 (16)	0.0574 (8)	
C35	0.4690 (3)	1.0902 (3)	0.28141 (15)	0.0490 (7)	
C36	0.3910 (4)	1.2112 (4)	0.2850 (2)	0.0646 (9)	
H9B	0.3647	1.2723	0.2457	0.078*	
C37	0.3538 (4)	1.2402 (5)	0.3447 (2)	0.0842 (13)	
H10B	0.3037	1.3207	0.3456	0.101*	
C38	0.3899 (4)	1.1507 (6)	0.4042 (2)	0.0883 (15)	
H11B	0.3622	1.1711	0.4447	0.106*	
C39	0.4654 (4)	1.0341 (5)	0.40307 (19)	0.0784 (12)	
H12B	0.4889	0.9751	0.4432	0.094*	
C40	0.6459 (4)	0.7965 (4)	0.07327 (17)	0.0671 (10)	
C41	0.7311 (4)	0.6765 (4)	0.1201 (2)	0.0778 (10)	
H14A	0.6651	0.6458	0.1444	0.093*	0.535 (4)
H14B	0.7704	0.6991	0.1523	0.093*	0.535 (4)
H14C	0.6961	0.6687	0.1651	0.093*	0.465 (4)
H14D	0.7984	0.7121	0.1174	0.093*	0.465 (4)
C42	0.8463 (8)	0.5647 (7)	0.1048 (4)	0.0778 (10)	0.535 (4)
C43	0.9800 (8)	0.5434 (7)	0.1138 (4)	0.072 (2)	0.535 (4)
H16B	1.0023	0.5938	0.1329	0.087*	0.535 (4)
C44	1.0858 (10)	0.4438 (8)	0.0940 (6)	0.082 (3)	0.535 (4)
H44	1.1765	0.4304	0.1001	0.098*	0.535 (4)
C45	1.0560 (10)	0.3701 (9)	0.0672 (4)	0.081 (3)	0.535 (4)
H18B	1.1263	0.3054	0.0551	0.097*	0.535 (4)
C46	0.9203 (9)	0.3882 (7)	0.0568 (4)	0.078 (2)	0.535 (4)
C47	0.8127 (8)	0.4873 (6)	0.0759 (3)	0.0697 (19)	0.535 (4)
C48	0.6745 (12)	0.5034 (9)	0.0661 (6)	0.1086 (19)	0.535 (4)
H21B	0.6032	0.5714	0.0745	0.130*	0.535 (4)
C49	0.6473 (13)	0.4207 (10)	0.0449 (7)	0.1086 (19)	0.535 (4)
H22B	0.5592	0.4220	0.0470	0.130*	0.535 (4)
C50	0.7563 (11)	0.3300 (9)	0.0188 (5)	0.1086 (19)	0.535 (4)
H23B	0.7362	0.2840	−0.0047	0.130*	0.535 (4)
C51	0.8885 (11)	0.3115 (9)	0.0284 (5)	0.1086 (19)	0.535 (4)
H24B	0.9583	0.2470	0.0158	0.130*	0.535 (4)
C42'	0.7119 (12)	0.5882 (10)	0.0827 (6)	0.1024 (14)	0.465 (4)
C43'	0.6485 (11)	0.5158 (10)	0.1209 (7)	0.1024 (14)	0.465 (4)
H16'	0.6118	0.5279	0.1620	0.123*	0.465 (4)
C44'	0.6408 (11)	0.4208 (10)	0.0953 (6)	0.1024 (14)	0.465 (4)
H17'	0.6018	0.3688	0.1212	0.123*	0.465 (4)
C45'	0.6911 (13)	0.4048 (14)	0.0316 (7)	0.1024 (14)	0.465 (4)
H18'	0.6783	0.3475	0.0144	0.123*	0.465 (4)
C46'	0.7591 (11)	0.4747 (10)	−0.0045 (6)	0.1024 (14)	0.465 (4)
C47'	0.7665 (11)	0.5695 (10)	0.0209 (6)	0.1024 (14)	0.465 (4)
C48'	0.8347 (11)	0.6422 (10)	−0.0192 (6)	0.1024 (14)	0.465 (4)
H21'	0.8411	0.7054	−0.0051	0.123*	0.465 (4)
C49'	0.8908 (14)	0.6185 (14)	−0.0786 (7)	0.1024 (14)	0.465 (4)
H22'	0.9348	0.6661	−0.1046	0.123*	0.465 (4)
C50'	0.8824 (14)	0.5247 (13)	−0.1000 (8)	0.1024 (14)	0.465 (4)
H23'	0.9236	0.5085	−0.1397	0.123*	0.465 (4)
C51'	0.8184 (11)	0.4587 (10)	−0.0659 (6)	0.1024 (14)	0.465 (4)
H24'	0.8118	0.3986	−0.0829	0.123*	0.465 (4)
C52	0.1214 (4)	1.0013 (4)	0.0845 (2)	0.0746 (12)	
H25B	0.1181	1.0735	0.0955	0.090*	
C53	−0.0098 (5)	0.8796 (5)	0.0961 (2)	0.0886 (14)	
H26D	0.0601	0.8406	0.0678	0.133*	
H26E	−0.0974	0.9067	0.0753	0.133*	
H26F	−0.0059	0.8230	0.1382	0.133*	
C54	−0.0924 (5)	1.0569 (6)	0.1441 (4)	0.143 (3)	
H27D	−0.0628	1.1158	0.1530	0.215*	
H27E	−0.1064	1.0064	0.1856	0.215*	
H27F	−0.1763	1.0981	0.1189	0.215*	

### Atomic displacement parameters (Å^2^)

**Table:**

	*U*^11^	*U*^22^	*U*^33^	*U*^12^	*U*^13^	*U*^23^
Cu1	0.02974 (17)	0.03313 (17)	0.04065 (19)	−0.01368 (13)	−0.00106 (13)	−0.01000 (13)
O1	0.0385 (11)	0.0370 (10)	0.0708 (14)	−0.0141 (9)	−0.0009 (10)	−0.0200 (10)
O2	0.0444 (12)	0.0598 (13)	0.0414 (11)	−0.0218 (10)	0.0018 (9)	−0.0085 (10)
O3	0.0453 (12)	0.0674 (14)	0.0395 (11)	−0.0258 (11)	0.0002 (9)	−0.0074 (10)
O4	0.0395 (11)	0.0347 (10)	0.0728 (15)	−0.0128 (9)	−0.0053 (10)	−0.0195 (10)
O5	0.0326 (10)	0.0517 (12)	0.0615 (13)	−0.0155 (9)	−0.0056 (9)	−0.0155 (10)
N1	0.0376 (13)	0.0526 (15)	0.0572 (16)	−0.0145 (12)	−0.0109 (12)	−0.0098 (12)
C1	0.0411 (16)	0.0341 (14)	0.0448 (16)	−0.0141 (12)	−0.0023 (12)	−0.0113 (12)
C2	0.0467 (17)	0.0347 (15)	0.077 (2)	−0.0139 (13)	−0.0058 (16)	−0.0184 (15)
C3	0.0483 (17)	0.0318 (14)	0.071 (2)	−0.0140 (13)	−0.0024 (15)	−0.0193 (14)
C4	0.058 (2)	0.0420 (17)	0.076 (2)	−0.0208 (15)	−0.0008 (17)	−0.0174 (16)
C5	0.055 (2)	0.066 (2)	0.098 (3)	−0.0314 (18)	0.002 (2)	−0.024 (2)
C6	0.052 (2)	0.072 (2)	0.102 (3)	−0.0239 (19)	−0.015 (2)	−0.030 (2)
C7	0.062 (2)	0.059 (2)	0.076 (3)	−0.0163 (18)	−0.0079 (19)	−0.0277 (19)
C8	0.0523 (19)	0.0468 (17)	0.076 (2)	−0.0185 (15)	0.0017 (17)	−0.0286 (17)
C9	0.084 (3)	0.086 (3)	0.074 (3)	−0.046 (2)	0.009 (2)	−0.029 (2)
C10	0.122 (4)	0.119 (4)	0.086 (3)	−0.071 (4)	0.018 (3)	−0.031 (3)
C11	0.134 (5)	0.117 (4)	0.069 (3)	−0.052 (4)	0.002 (3)	−0.020 (3)
C12	0.091 (3)	0.097 (3)	0.089 (3)	−0.030 (3)	−0.020 (3)	−0.032 (3)
C13	0.0501 (18)	0.0445 (16)	0.0413 (16)	−0.0262 (14)	0.0009 (14)	−0.0101 (13)
C14	0.067 (2)	0.079 (2)	0.0399 (17)	−0.0407 (19)	−0.0023 (15)	−0.0066 (16)
C15	0.0529 (18)	0.0597 (19)	0.0338 (15)	−0.0212 (16)	−0.0022 (14)	0.0013 (14)
C16	0.091 (3)	0.087 (3)	0.043 (2)	−0.025 (2)	0.011 (2)	−0.0122 (19)
C17	0.083 (3)	0.146 (5)	0.060 (3)	−0.016 (4)	0.029 (2)	−0.008 (3)
C18	0.063 (3)	0.149 (5)	0.072 (3)	−0.046 (3)	0.004 (2)	0.036 (3)
C19	0.063 (2)	0.090 (3)	0.066 (2)	−0.042 (2)	−0.020 (2)	0.031 (2)
C20	0.0502 (19)	0.058 (2)	0.0472 (18)	−0.0207 (16)	−0.0111 (15)	0.0117 (15)
C21	0.082 (3)	0.052 (2)	0.075 (3)	−0.018 (2)	−0.008 (2)	−0.0012 (19)
C22	0.157 (6)	0.056 (3)	0.116 (4)	−0.025 (3)	−0.029 (4)	−0.010 (3)
C23	0.212 (9)	0.073 (4)	0.160 (7)	−0.081 (5)	−0.068 (6)	0.027 (4)
C24	0.120 (5)	0.106 (5)	0.129 (5)	−0.080 (4)	−0.053 (4)	0.049 (4)
C25	0.0348 (15)	0.0483 (17)	0.0558 (19)	−0.0123 (13)	−0.0044 (14)	−0.0098 (14)
C26	0.0378 (18)	0.063 (2)	0.091 (3)	−0.0095 (16)	−0.0100 (17)	−0.012 (2)
C27	0.069 (3)	0.094 (3)	0.089 (3)	−0.027 (2)	−0.022 (2)	−0.035 (2)
Cu2	0.0715 (3)	0.1253 (4)	0.0355 (2)	−0.0693 (3)	0.00152 (19)	−0.0063 (2)
O6	0.0740 (17)	0.105 (2)	0.0453 (13)	−0.0516 (16)	−0.0009 (12)	−0.0139 (13)
O7	0.0636 (16)	0.099 (2)	0.0579 (15)	−0.0423 (15)	0.0122 (12)	−0.0134 (14)
O8	0.113 (2)	0.173 (3)	0.0461 (15)	−0.106 (2)	0.0112 (15)	−0.0278 (17)
O9	0.0752 (18)	0.150 (3)	0.0545 (15)	−0.072 (2)	−0.0109 (13)	0.0065 (17)
O10	0.0654 (17)	0.114 (2)	0.0606 (16)	−0.0584 (17)	0.0033 (13)	−0.0037 (15)
N2	0.0477 (17)	0.074 (2)	0.081 (2)	−0.0245 (15)	−0.0078 (15)	−0.0046 (17)
C28	0.087 (3)	0.094 (3)	0.0348 (18)	−0.053 (2)	−0.0097 (18)	−0.0002 (17)
C29	0.095 (3)	0.078 (3)	0.046 (2)	−0.041 (2)	−0.0122 (19)	−0.0057 (18)
C30	0.0500 (18)	0.063 (2)	0.0435 (17)	−0.0241 (16)	−0.0039 (14)	−0.0108 (15)
C31	0.065 (2)	0.077 (3)	0.059 (2)	−0.015 (2)	−0.0025 (18)	−0.028 (2)
C32	0.079 (3)	0.060 (2)	0.085 (3)	−0.006 (2)	−0.021 (2)	−0.016 (2)
C33	0.085 (3)	0.072 (3)	0.060 (2)	−0.030 (2)	−0.018 (2)	0.006 (2)
C34	0.055 (2)	0.078 (2)	0.0443 (18)	−0.0350 (18)	−0.0006 (15)	−0.0084 (16)
C35	0.0386 (16)	0.065 (2)	0.0467 (17)	−0.0228 (15)	−0.0004 (13)	−0.0152 (15)
C36	0.051 (2)	0.076 (2)	0.067 (2)	−0.0173 (18)	−0.0099 (17)	−0.0249 (19)
C37	0.048 (2)	0.110 (3)	0.100 (4)	−0.012 (2)	−0.004 (2)	−0.060 (3)
C38	0.055 (2)	0.152 (5)	0.070 (3)	−0.038 (3)	0.014 (2)	−0.055 (3)
C39	0.075 (3)	0.127 (4)	0.046 (2)	−0.057 (3)	0.0077 (19)	−0.017 (2)
C40	0.076 (3)	0.100 (3)	0.0392 (18)	−0.044 (2)	0.0075 (17)	−0.0273 (19)
C41	0.072 (2)	0.073 (2)	0.086 (3)	−0.0228 (19)	0.0066 (19)	−0.026 (2)
C42	0.072 (2)	0.073 (2)	0.086 (3)	−0.0228 (19)	0.0066 (19)	−0.026 (2)
C43	0.074 (5)	0.071 (5)	0.060 (4)	−0.022 (4)	−0.007 (4)	−0.001 (3)
C44	0.075 (6)	0.053 (5)	0.111 (7)	−0.021 (4)	−0.006 (5)	−0.013 (5)
C45	0.080 (7)	0.073 (5)	0.061 (5)	−0.005 (5)	0.015 (5)	−0.013 (4)
C46	0.110 (7)	0.056 (4)	0.051 (4)	−0.018 (4)	−0.006 (4)	−0.004 (3)
C47	0.081 (5)	0.056 (4)	0.054 (4)	−0.013 (4)	0.000 (4)	−0.007 (3)
C48	0.132 (5)	0.081 (3)	0.117 (4)	−0.036 (3)	−0.021 (4)	−0.030 (3)
C49	0.132 (5)	0.081 (3)	0.117 (4)	−0.036 (3)	−0.021 (4)	−0.030 (3)
C50	0.132 (5)	0.081 (3)	0.117 (4)	−0.036 (3)	−0.021 (4)	−0.030 (3)
C51	0.132 (5)	0.081 (3)	0.117 (4)	−0.036 (3)	−0.021 (4)	−0.030 (3)
C42'	0.090 (3)	0.082 (3)	0.114 (3)	−0.0068 (19)	−0.019 (2)	−0.024 (2)
C43'	0.090 (3)	0.082 (3)	0.114 (3)	−0.0068 (19)	−0.019 (2)	−0.024 (2)
C44'	0.090 (3)	0.082 (3)	0.114 (3)	−0.0068 (19)	−0.019 (2)	−0.024 (2)
C45'	0.090 (3)	0.082 (3)	0.114 (3)	−0.0068 (19)	−0.019 (2)	−0.024 (2)
C46'	0.090 (3)	0.082 (3)	0.114 (3)	−0.0068 (19)	−0.019 (2)	−0.024 (2)
C47'	0.090 (3)	0.082 (3)	0.114 (3)	−0.0068 (19)	−0.019 (2)	−0.024 (2)
C48'	0.090 (3)	0.082 (3)	0.114 (3)	−0.0068 (19)	−0.019 (2)	−0.024 (2)
C49'	0.090 (3)	0.082 (3)	0.114 (3)	−0.0068 (19)	−0.019 (2)	−0.024 (2)
C50'	0.090 (3)	0.082 (3)	0.114 (3)	−0.0068 (19)	−0.019 (2)	−0.024 (2)
C51'	0.090 (3)	0.082 (3)	0.114 (3)	−0.0068 (19)	−0.019 (2)	−0.024 (2)
C52	0.059 (2)	0.066 (2)	0.091 (3)	−0.032 (2)	−0.019 (2)	0.011 (2)
C53	0.083 (3)	0.123 (4)	0.080 (3)	−0.069 (3)	−0.002 (2)	−0.011 (3)
C54	0.068 (3)	0.132 (5)	0.221 (8)	−0.018 (3)	0.027 (4)	−0.071 (5)

### Geometric parameters (Å, º)

**Table:**

Cu1—O1	1.9501 (19)	O9—Cu2^ii^	1.974 (3)
Cu1—O4^i^	1.9578 (18)	O10—C52	1.230 (5)
Cu1—O2	1.972 (2)	N2—C52	1.312 (5)
Cu1—O3^i^	1.980 (2)	N2—C53	1.439 (5)
Cu1—O5	2.1535 (19)	N2—C54	1.452 (6)
Cu1—Cu1^i^	2.6485 (6)	C28—C29	1.516 (5)
O1—C1	1.253 (3)	C29—C30	1.516 (5)
O2—C13	1.254 (4)	C29—H2B1	0.9700
O3—C13	1.251 (4)	C29—H2B2	0.9700
O3—Cu1^i^	1.980 (2)	C30—C31	1.361 (5)
O4—C1	1.251 (3)	C30—C35	1.422 (4)
O4—Cu1^i^	1.9578 (18)	C31—C32	1.407 (6)
O5—C25	1.236 (4)	C31—H4B	0.9300
N1—C25	1.309 (4)	C32—C33	1.349 (6)
N1—C26	1.444 (4)	C32—H5B	0.9300
N1—C27	1.457 (4)	C33—C34	1.395 (5)
C1—C2	1.514 (4)	C33—H6B	0.9300
C2—C3	1.501 (4)	C34—C39	1.416 (5)
C2—H2A	0.9700	C34—C35	1.428 (5)
C2—H2B	0.9700	C35—C36	1.415 (5)
C3—C4	1.365 (5)	C36—C37	1.362 (5)
C3—C8	1.413 (5)	C36—H9B	0.9300
C4—C5	1.405 (5)	C37—C38	1.391 (7)
C4—H4	0.9300	C37—H10B	0.9300
C5—C6	1.343 (6)	C38—C39	1.352 (7)
C5—H5	0.9300	C38—H11B	0.9300
C6—C7	1.407 (6)	C39—H12B	0.9300
C6—H6	0.9300	C40—C41	1.521 (6)
C7—C12	1.409 (6)	C41—C42	1.532 (8)
C7—C8	1.424 (5)	C41—C42'	1.575 (12)
C8—C9	1.414 (5)	C41—H14A	0.9700
C9—C10	1.351 (6)	C41—H14B	0.9700
C9—H9	0.9300	C41—H14C	0.9700
C10—C11	1.390 (7)	C41—H14D	0.9700
C10—H10	0.9300	C42—C43	1.365 (11)
C11—C12	1.361 (7)	C42—C47	1.418 (10)
C11—H11	0.9300	C43—C44	1.431 (13)
C12—H12	0.9300	C43—H16B	0.9300
C13—C14	1.519 (4)	C44—C45	1.329 (13)
C14—C15	1.498 (4)	C44—H44	0.9300
C14—H14E	0.9700	C45—C46	1.403 (11)
C14—H14F	0.9700	C45—H18B	0.9300
C15—C16	1.361 (5)	C46—C51	1.394 (12)
C15—C20	1.419 (5)	C46—C47	1.429 (10)
C16—C17	1.402 (7)	C47—C48	1.434 (13)
C16—H16A	0.9300	C48—C49	1.348 (15)
C17—C18	1.347 (8)	C48—H21B	0.9300
C17—H17A	0.9300	C49—C50	1.444 (12)
C18—C19	1.393 (7)	C49—H22B	0.9300
C18—H18A	0.9300	C50—C51	1.361 (12)
C19—C20	1.417 (5)	C50—H23B	0.9300
C19—C24	1.420 (8)	C51—H24B	0.9300
C20—C21	1.412 (5)	C42'—C43'	1.377 (16)
C21—C22	1.369 (6)	C42'—C47'	1.399 (16)
C21—H21A	0.9300	C43'—C44'	1.442 (16)
C22—C23	1.396 (10)	C43'—H16'	0.9300
C22—H22A	0.9300	C44'—C45'	1.416 (14)
C23—C24	1.321 (10)	C44'—H17'	0.9300
C23—H23A	0.9300	C45'—C46'	1.375 (18)
C24—H24A	0.9300	C45'—H18'	0.9300
C25—H25A	0.9695	C46'—C51'	1.399 (13)
C26—H26A	0.9600	C46'—C47'	1.437 (15)
C26—H26B	0.9600	C47'—C48'	1.430 (13)
C26—H26C	0.9600	C48'—C49'	1.373 (14)
C27—H27A	0.9600	C48'—H21'	0.9300
C27—H27B	0.9600	C49'—C50'	1.380 (15)
C27—H27C	0.9600	C49'—H22'	0.9300
Cu2—O8^ii^	1.962 (3)	C50'—C51'	1.297 (14)
Cu2—O7	1.967 (3)	C50'—H23'	0.9300
Cu2—O6	1.970 (3)	C51'—H24'	0.9300
Cu2—O9^ii^	1.974 (3)	C52—H25B	0.9636
Cu2—O10	2.143 (2)	C53—H26D	0.9600
Cu2—Cu2^ii^	2.6455 (8)	C53—H26E	0.9600
O6—C28	1.254 (4)	C53—H26F	0.9600
O7—C40	1.252 (5)	C54—H27D	0.9600
O8—C28	1.251 (5)	C54—H27E	0.9600
O8—Cu2^ii^	1.962 (3)	C54—H27F	0.9600
O9—C40	1.253 (5)		
			
O1—Cu1—O4^i^	167.77 (8)	O8—C28—O6	126.3 (4)
O1—Cu1—O2	89.29 (9)	O8—C28—C29	117.5 (3)
O4^i^—Cu1—O2	89.56 (9)	O6—C28—C29	116.1 (4)
O1—Cu1—O3^i^	89.33 (9)	C28—C29—C30	113.0 (3)
O4^i^—Cu1—O3^i^	89.23 (9)	C28—C29—H2B1	109.0
O2—Cu1—O3^i^	167.86 (8)	C30—C29—H2B1	109.0
O1—Cu1—O5	98.64 (8)	C28—C29—H2B2	109.0
O4^i^—Cu1—O5	93.52 (8)	C30—C29—H2B2	109.0
O2—Cu1—O5	101.74 (8)	H2B1—C29—H2B2	107.8
O3^i^—Cu1—O5	90.39 (8)	C31—C30—C35	118.9 (3)
O1—Cu1—Cu1^i^	86.47 (6)	C31—C30—C29	121.7 (3)
O4^i^—Cu1—Cu1^i^	81.31 (6)	C35—C30—C29	119.5 (3)
O2—Cu1—Cu1^i^	86.99 (6)	C30—C31—C32	122.0 (4)
O3^i^—Cu1—Cu1^i^	80.89 (6)	C30—C31—H4B	119.0
O5—Cu1—Cu1^i^	169.88 (6)	C32—C31—H4B	119.0
C1—O1—Cu1	120.12 (17)	C33—C32—C31	119.7 (4)
C13—O2—Cu1	119.63 (19)	C33—C32—H5B	120.1
C13—O3—Cu1^i^	126.54 (19)	C31—C32—H5B	120.1
C1—O4—Cu1^i^	125.86 (18)	C32—C33—C34	121.2 (4)
C25—O5—Cu1	117.18 (19)	C32—C33—H6B	119.4
C25—N1—C26	120.9 (3)	C34—C33—H6B	119.4
C25—N1—C27	122.2 (3)	C33—C34—C39	122.5 (4)
C26—N1—C27	116.9 (3)	C33—C34—C35	119.3 (3)
O4—C1—O1	126.2 (2)	C39—C34—C35	118.2 (4)
O4—C1—C2	117.2 (2)	C36—C35—C30	123.3 (3)
O1—C1—C2	116.5 (2)	C36—C35—C34	117.8 (3)
C3—C2—C1	113.8 (2)	C30—C35—C34	118.9 (3)
C3—C2—H2A	108.8	C37—C36—C35	121.4 (4)
C1—C2—H2A	108.8	C37—C36—H9B	119.3
C3—C2—H2B	108.8	C35—C36—H9B	119.3
C1—C2—H2B	108.8	C36—C37—C38	120.7 (4)
H2A—C2—H2B	107.7	C36—C37—H10B	119.6
C4—C3—C8	119.2 (3)	C38—C37—H10B	119.6
C4—C3—C2	120.2 (3)	C39—C38—C37	119.8 (4)
C8—C3—C2	120.6 (3)	C39—C38—H11B	120.1
C3—C4—C5	121.3 (4)	C37—C38—H11B	120.1
C3—C4—H4	119.3	C38—C39—C34	122.0 (4)
C5—C4—H4	119.3	C38—C39—H12B	119.0
C6—C5—C4	120.4 (4)	C34—C39—H12B	119.0
C6—C5—H5	119.8	O7—C40—O9	125.7 (4)
C4—C5—H5	119.8	O7—C40—C41	117.0 (4)
C5—C6—C7	121.0 (3)	O9—C40—C41	117.3 (4)
C5—C6—H6	119.5	C40—C41—C42	130.0 (5)
C7—C6—H6	119.5	C40—C41—C42'	98.4 (5)
C6—C7—C12	122.9 (4)	C42—C41—C42'	55.2 (5)
C6—C7—C8	118.6 (4)	C40—C41—H14A	104.8
C12—C7—C8	118.4 (4)	C42—C41—H14A	104.8
C3—C8—C9	122.8 (3)	C42'—C41—H14A	72.1
C3—C8—C7	119.4 (3)	C40—C41—H14B	104.8
C9—C8—C7	117.7 (4)	C42—C41—H14B	104.8
C10—C9—C8	122.3 (4)	C42'—C41—H14B	156.4
C10—C9—H9	118.9	H14A—C41—H14B	105.8
C8—C9—H9	118.9	C40—C41—H14C	112.0
C9—C10—C11	119.7 (5)	C42—C41—H14C	117.4
C9—C10—H10	120.1	C42'—C41—H14C	113.7
C11—C10—H10	120.1	H14A—C41—H14C	44.3
C12—C11—C10	120.6 (5)	H14B—C41—H14C	61.5
C12—C11—H11	119.7	C40—C41—H14D	85.2
C10—C11—H11	119.7	C42—C41—H14D	87.2
C11—C12—C7	121.3 (4)	C42'—C41—H14D	133.4
C11—C12—H12	119.4	H14A—C41—H14D	151.9
C7—C12—H12	119.4	H14B—C41—H14D	46.2
O3—C13—O2	125.9 (3)	H14C—C41—H14D	107.5
O3—C13—C14	115.4 (3)	C43—C42—C47	119.7 (7)
O2—C13—C14	118.6 (3)	C43—C42—C41	121.4 (7)
C15—C14—C13	117.5 (3)	C47—C42—C41	118.7 (7)
C15—C14—H14E	107.9	C42—C43—C44	120.3 (8)
C13—C14—H14E	107.9	C42—C43—H16B	119.8
C15—C14—H14F	107.9	C44—C43—H16B	119.8
C13—C14—H14F	107.9	C45—C44—C43	120.8 (9)
H14E—C14—H14F	107.2	C45—C44—H44	119.6
C16—C15—C20	119.2 (3)	C43—C44—H44	119.6
C16—C15—C14	120.0 (4)	C44—C45—C46	121.1 (8)
C20—C15—C14	120.7 (3)	C44—C45—H18B	119.4
C15—C16—C17	121.0 (5)	C46—C45—H18B	119.4
C15—C16—H16A	119.5	C45—C46—C51	121.4 (9)
C17—C16—H16A	119.5	C45—C46—C47	119.2 (8)
C18—C17—C16	120.5 (5)	C51—C46—C47	119.5 (9)
C18—C17—H17A	119.8	C42—C47—C48	122.5 (7)
C16—C17—H17A	119.8	C42—C47—C46	119.0 (8)
C17—C18—C19	120.9 (4)	C48—C47—C46	118.6 (8)
C17—C18—H18A	119.5	C49—C48—C47	120.5 (10)
C19—C18—H18A	119.5	C49—C48—H21B	119.8
C18—C19—C20	119.1 (4)	C47—C48—H21B	119.8
C18—C19—C24	122.3 (5)	C48—C49—C50	119.2 (12)
C20—C19—C24	118.6 (5)	C48—C49—H22B	120.4
C21—C20—C19	117.8 (4)	C50—C49—H22B	120.4
C21—C20—C15	123.0 (3)	C51—C50—C49	120.2 (10)
C19—C20—C15	119.2 (4)	C51—C50—H23B	119.9
C22—C21—C20	120.6 (5)	C49—C50—H23B	119.9
C22—C21—H21A	119.7	C50—C51—C46	120.7 (10)
C20—C21—H21A	119.7	C50—C51—H24B	119.6
C21—C22—C23	121.0 (6)	C46—C51—H24B	119.6
C21—C22—H22A	119.5	C43'—C42'—C47'	120.6 (11)
C23—C22—H22A	119.5	C43'—C42'—C41	113.0 (10)
C24—C23—C22	119.7 (6)	C47'—C42'—C41	126.0 (11)
C24—C23—H23A	120.1	C42'—C43'—C44'	118.0 (12)
C22—C23—H23A	120.1	C42'—C43'—H16'	121.0
C23—C24—C19	122.3 (6)	C44'—C43'—H16'	121.0
C23—C24—H24A	118.9	C45'—C44'—C43'	121.5 (12)
C19—C24—H24A	118.9	C45'—C44'—H17'	119.2
O5—C25—N1	125.4 (3)	C43'—C44'—H17'	119.2
O5—C25—H25A	122.7	C46'—C45'—C44'	119.5 (13)
N1—C25—H25A	111.9	C46'—C45'—H18'	120.3
N1—C26—H26A	109.5	C44'—C45'—H18'	120.3
N1—C26—H26B	109.5	C45'—C46'—C51'	122.5 (12)
H26A—C26—H26B	109.5	C45'—C46'—C47'	118.8 (11)
N1—C26—H26C	109.5	C51'—C46'—C47'	118.7 (11)
H26A—C26—H26C	109.5	C42'—C47'—C48'	121.6 (11)
H26B—C26—H26C	109.5	C42'—C47'—C46'	121.4 (11)
N1—C27—H27A	109.5	C48'—C47'—C46'	116.9 (11)
N1—C27—H27B	109.5	C49'—C48'—C47'	119.9 (12)
H27A—C27—H27B	109.5	C49'—C48'—H21'	120.1
N1—C27—H27C	109.5	C47'—C48'—H21'	120.1
H27A—C27—H27C	109.5	C48'—C49'—C50'	120.7 (14)
H27B—C27—H27C	109.5	C48'—C49'—H22'	119.7
O8^ii^—Cu2—O7	89.67 (14)	C50'—C49'—H22'	119.7
O8^ii^—Cu2—O6	168.00 (11)	C51'—C50'—C49'	121.4 (16)
O7—Cu2—O6	89.60 (11)	C51'—C50'—H23'	119.3
O8^ii^—Cu2—O9^ii^	89.22 (14)	C49'—C50'—H23'	119.3
O7—Cu2—O9^ii^	167.85 (11)	C50'—C51'—C46'	122.4 (13)
O6—Cu2—O9^ii^	88.98 (13)	C50'—C51'—H24'	118.8
O8^ii^—Cu2—O10	96.72 (12)	C46'—C51'—H24'	118.8
O7—Cu2—O10	95.71 (11)	O10—C52—N2	125.3 (4)
O6—Cu2—O10	95.27 (11)	O10—C52—H25B	125.7
O9^ii^—Cu2—O10	96.44 (11)	N2—C52—H25B	109.0
O8^ii^—Cu2—Cu2^ii^	85.27 (8)	N2—C53—H26D	109.5
O7—Cu2—Cu2^ii^	83.41 (8)	N2—C53—H26E	109.5
O6—Cu2—Cu2^ii^	82.75 (7)	H26D—C53—H26E	109.5
O9^ii^—Cu2—Cu2^ii^	84.44 (8)	N2—C53—H26F	109.5
O10—Cu2—Cu2^ii^	177.82 (8)	H26D—C53—H26F	109.5
C28—O6—Cu2	124.0 (3)	H26E—C53—H26F	109.5
C40—O7—Cu2	124.0 (3)	N2—C54—H27D	109.5
C28—O8—Cu2^ii^	121.6 (2)	N2—C54—H27E	109.5
C40—O9—Cu2^ii^	122.4 (3)	H27D—C54—H27E	109.5
C52—O10—Cu2	123.1 (3)	N2—C54—H27F	109.5
C52—N2—C53	120.1 (4)	H27D—C54—H27F	109.5
C52—N2—C54	123.3 (4)	H27E—C54—H27F	109.5
C53—N2—C54	116.5 (4)		

Symmetry codes: (i) −*x*+2, −*y*+1, −*z*+1; (ii) −*x*+1, −*y*+2, −*z*.

## References

[bb1] Bruker (2007). *APEX2* and *SAINT* Bruker AXS Inc., Madison, Wisconsin, USA.

[bb2] Chen, L.-F., Zhang, J., Song, L.-J., Wang, W.-G. & Ju, Z.-F. (2004). *Acta Cryst.* E**60**, m1032–m1034.

[bb3] Ji, L.-L., Liu, J.-S. & Song, W.-D. (2011). *Acta Cryst.* E**67**, m606. 10.1107/S1600536811013353 PMC3089144 21754324

[bb4] Sheldrick, G. M. (1996). *SADABS* University of Göttingen, Germany.

[bb5] Sheldrick, G. M. (2008). *Acta Cryst.* A**64**, 112–122. 10.1107/S0108767307043930 18156677

[bb6] Xia, H.-T., Liu, Y.-F. & Li, S.-A. (2006). *Acta Cryst.* E**62**, m2653–m2655.

[bb7] Yang, Y.-Q., Li, C.-H. L. W. & Kuang, Y.-F. (2008). *Chin. J. Struct. Chem.* **30**, 4524–4530.

[bb8] Yin, F.-J., Zhao, H. & Hu, X.-L. (2010). *Synth. React. Inorg. Met. Org. Nano-Met. Chem.* **40**, 606–612.

